# Patient and public involvement workshop to shape artificial intelligence-supported connected asthma self-management research

**DOI:** 10.1371/journal.pdig.0000521

**Published:** 2024-05-30

**Authors:** Chi Yan Hui, Ann Victoria Shenton, Claire Martin, David Weatherill, Dianna Moylan, Morag Hayes, Laura Gonzalez Rienda, Emma Kinley, Stefanie Eck, Hilary Pinnock

**Affiliations:** 1 Asthma UK Centre for Applied Research, Usher Institute, University of Edinburgh, United Kingdom; 2 School of Psychology, Faculty of Health, Liverpool John Moore’s University, United Kingdom; 3 Institute of General Practice and Health Services Research, TUM School of Medicine, Technical University of Munich (TUM), Germany; Iran University of Medical Sciences, IRAN (ISLAMIC REPUBLIC OF)

## Abstract

Digital interventions with artificial intelligence (AI) can potentially support people with asthma to reduce the risk of exacerbation. Engaging patients throughout the development process is essential to ensure usability of the intervention for the end-users. Using our Connected for Asthma (C4A) intervention as an exemplar, we explore how patient involvement can shape a digital intervention. Seven Patient and Public Involvement (PPI) colleagues from the Asthma UK Centre for Applied Research participated in four advisory workshops to discuss how they would prefer to use/interact with AI to support living with their asthma, the benefit and caveats to use the AI that incorporated asthma monitoring and indoor/outdoor environmental data. Discussion focussed on the three most wanted use cases identified in our previous studies. PPI colleagues wanted AI to support data collection, remind them about self-management tasks, teach them about asthma environmental triggers, identify risk, and empower them to confidently look after their asthma whilst emphasising that AI does not replace clinicians. The discussion informed the key components in the next C4A interventions, including the approach to interacting with AI, the technology features and the research topics. Attendees highlighted the importance of considering health inequities, the presentation of data, and concerns about data accuracy, data privacy, security and ownership. We have demonstrated how patient roles can shift from that of ‘user’ (the traditional ‘tester’ of a digital intervention), to a co-design partner who shapes the next iteration of the intervention. Technology innovators should seek practical and feasible strategies to involve PPI colleagues throughout the development cycle of a digital intervention; supporting researchers to explore the barriers, concerns, enablers and advantages of implementing digital healthcare.

## Author summary

Artificial intelligence (AI) can help people with asthma to manage their condition better. We invited seven people living with asthma from the Asthma UK Centre for Applied Research Patient and Public Involvement (PPI) group to join four workshops. The PPI colleagues shared their opinions and ideas about the future AI self-management tools linking asthma control and environmental factors. They discussed what they wanted AI to do for them in an indoor connected system, an outdoor smart city, and how they wanted to interact with the information. They highlighted some important issues, such as fairness, data quality, and privacy. These ideas will be used to co-design our future research. The workshop showed how involving people with asthma early in the AI design process can direct future research and potentially make the digital tool more relevant and effective for them. In this way, people who live with a condition such as asthma on a day-to-day basis can be a co-design partner to help shape future AI.

## Introduction

### Asthma self-management, air quality and technology

In the UK, 4.3 million people of all ages, are living with asthma. Asthma is a variable condition, triggered by viral infections, exercise, allergens (including pollens), air temperature, as well as a range of indoor/outdoor air pollutants [1,2]. People with asthma need to know how to reduce exposure to triggers, recognise deterioration, and adjust their treatment to respond to changes in their asthma status. They need to balance the restrictions of avoiding triggers with the benefits of being physically active and enjoying meaningful activities and lifestyle, contributing to their mental wellbeing as well as protecting lung function. Supported by their healthcare professionals (HCPs), self-management is a highly effective strategy for reducing the risk of attacks and improving control [3].

The health risks of poor air quality and its effect on asthma patients is increasingly being recognised [1,2,4]. Technology can enable informed decision-making and support patients to balance the individualised risk of trigger exposure with participating in the paid work, leisure activities and exercise that they value.

Internet-of-things (IoT) environmental sensors can detect an increase in known risk factors (e.g. environmental pollen) and, combined with other asthma self-management devices (e.g. smart spirometers and smart inhalers), can form a connected system that can provide early warning of an attack. With this prediction, the AI can provide timely advice to patients on adjusting their medication to prevent the attack progressing. Where appropriate, the system can also connect patients to their clinicians for further advice.

Digital health, including ehealth/mhealth, are increasingly seen as able to support effective self-management. Information and communication technology (ICT) models are gradually transforming from standalone systems, that provide healthcare information on a dashboard, into two-way, AI-enabled systems with multiple connected sensors to provide holistic self-management support in self-diagnosing, monitoring triggers, and attack prediction [5,6]. This digital transformation evolved rapidly during the COVID pandemic; as connected systems allowed home surveillance of COVID infections avoiding the risk of viral spread during a visit to the clinician [7]. However, the question remains how such data/AI-enabled digital health applications maybe valued by the end users (that is, the patients), and how to increase engagement and use of these technologies [8].

### The role of patient involvement in digital health care research and development

Despite promotion of patient and public involvement (PPI) [9,10], in most digital intervention, patients’ involvement is typically recognised as a tester to evaluate the usability/health outcomes of a digital service, with the aim of supporting the researcher to generate evidence for further development and roll out. Patient involvement at the conceptual stage of shaping innovation is limited despite potential advantages of early involvement [11]. Firstly, providing co-design opportunities for patients to shape a technology pre-development ensures the resulting intervention is of interest and useful to them. Secondly, research participants are typically positive about ready-made interventions and studies of digital technologies rarely garner negative comments [12]. PPI can consult patients about future developments, when patients may be more willing to provide critical and innovative feedback.

Finally, PPI enables researchers to hear the patients’ voice not as research participants but as colleagues helping to shape research. Reflecting this, five of the authors on this paper are the PPI colleagues who contributed to the workshops. Despite promotion of this collaborative process [13], and recognition of the benefits, there is little published work reporting on the role of PPI in shaping the future direction of a digital intervention, and the design of future research [14].

### Patient involvement in the Connected for Asthma research

The Connected for Asthma (C4A) research programme has involved patients (and other stakeholders) at every stage of the research phase, to co-create a digital intervention to support asthma self-management.

From our previous PPI discussions, we know patients and clinicians want to monitor their asthma and their triggers and want timely feedback to support their care, within a connected ‘super-app’ that can connect them with their clinicians. They believe AI could be helpful to provide advice in the connected system, though the AI has to be trustworthy and reliable in order to encourage adoption [15]. We summarised their preferred use of AI in three user cases to be discussed in the workshop.

In this paper, we report PPI advisory workshops conducted within the C4A study as an exemplar of empowering patients’ role in shaping innovation towards a digital intervention that would wish to adopt and use in real life.

## Methods

### Ethics statement

The University of Edinburgh Medical Research Ethics Committee confirmed that this was a consultation exercise with PPI colleagues designed to shape a research agenda, so that ethical approval and formal consent was not required (REC Reference: 22-EMREC-057). Five of the PPI colleagues are co-authors, and two chose not to be named. We followed the standards of the National Institute for Health Research (NIHR) for Patient and Public Involvement and Greenhalgh et al ‘s PPI framework to shape the work [13,16].

### Workshop design

We used the key learnings from our previous studies to design the workshops [17]:

- Most patients have heard of AI but few know what it is, and the range of its capabilities.
- Patients have different ways to manage their asthma, and they develop their own self-management routine and that influences if/how they would like to use technology to look after their asthma.
- Building on existing proven technologies, and research identifying patients’ most ‘wanted’ features in related to the Al and environmental data [17], we determined three potential use-cases that may have future utility.

### Aim of the workshop

To shape the technology features and research topics of the next digital intervention in the C4A portfolio. To inform which of the use-cases had utility, we aimed to consult PPI colleagues on how they would prefer to use AI including indoor/outdoor environmental data to support their self-management and more generally, the workshop aimed to explore how they PPI wish to interact with AI?

### PPI colleagues’ invitations

We invited PPI colleagues from the Asthma UK Centre for Applied Research (AUKCAR) to join a workshop via a post in the Centre’s Twitter. The post contained information such as the aim, time/location of the workshop and reimbursement. Information was also sent to previous C4A study participants who had given consent to be contacted about future activities.

### Procedure

To accommodate availability, we carried out four advisory workshops/discussions with seven PPI colleagues, who were adults with asthma, with variable experience of using technology to support their asthma.

In each workshop, a researcher (CYH) led the discussion supported by up to two PPI facilitators (LGR, and/or EK, and/or SE). We ran face-to-face workshops on the University premises with an online option for PPI colleagues unable to travel to the university to participate. To stimulate ideas about their self-management routine and how AI and environmental data could support their daily self-management tasks, we sent PPI colleagues a table to act as a framework to structure the thoughts and experiences we wanted to discuss in the workshop (see S1 Table, self-management table).

Each workshop ran for one hour and consisted of an introduction, two exercises to stimulate discussion and a final ‘wrap-up’ (See Table 1). During the workshop, the PPI facilitators noted the key points of the discussion, and raised questions if, for example, they felt an explanation would be helpful for any technical concepts to ensure full understanding for contributing PPI members. The discussions were recorded to aid notetaking and enable key points to be summarised, but the conversations were not transcribed and analysed thematically as would be expected in qualitative research. This is in line with the principles of working with colleagues [13,16].

**Table 1:** The workshop procedure.

	Aim of the session	Process
Introductions	The researcher (CYP), PPI facilitators introduced themselves and explained the purpose of the workshop in their introductions. PPI colleagues described their prior experience and understanding of using technologies (or not).	The researcher used lay language supported by graphics and PowerPoint slides to introduce basic information about AI, indoor and outdoor environmental monitoring. PPI colleagues were encouraged to provide both positive and negative feedback throughout the discussion.
Exercise one	The researcher invited PPI colleagues to present their thoughts on how they manage their asthma, and how environmental data and AI could support daily self-management tasks	Group discussion was invited. Positive and negative ideas that had emerged in the previous workshops were brought into the discussion by the researcher to enhance the discussion
Exercise two	The researcher led a discussion on the three potential use cases (derived from our previous work) of AI, indoor and outdoor environmental data	The researcher presented the three potential user cases and encouraged discussion on whether the technologies would be of benefit to them as people with asthma, how they would like to interact with the AI within those use cases, and if they had any concerns about using such technologies.
Wrap up and follow up	The researcher summarised the discussion, invited PPI colleagues to reflect on what they had suggested and provide any feedback on how the workshop could be improved.	The researcher outlined how the discussions would be used to shape the next digital intervention and asked if they wished to be part of follow up activities (including as an author on a publication)

### Reporting

We adopted NIHR Guidance for Reporting Involvement of Patients and the Public 2(GRIPP2) checklist to report the PPI involvement in manuscript (see Table 2) [18]. All participants were sent the report so that they could review the conclusions and add any additional points.

**Table 2:** Reporting Involvement of Patients and the Public 2(GRIPP2) checklist.

GRIPP2 reporting checklist [ref]	Reported in the manuscript
Aim	Introduction and methods sections (page 3–5)
Methods	Methods section (page 4–5)
Results (both positive and negative)	Result section (page 6–8)
Discussion	Discussion section (page 8–10)
Reflections	Reflections, challenges, and limitations section (page 10–11)

## Results

A total of seven PPI colleagues joined two PPI facilitators and the researcher in the workshop discussions. Experience with technology ranged from innovator (who may not have a clear ideas on how to use AI), early adopter (who has some AI experiences that learnt from public media, or through PPI research activities), to expertise in data analysis [19], but we did not sample colleagues based on the range of technology/AI experience. Because of challenges of finding a time when all participants were free during the limited timescale of the project, we opted to run four sessions to enable all the PPI colleagues to contribute, but ensured that themes from one discussion were fed into the next to enable some continuity of discussion.

### Discussion on exercise 1: How can AI, indoor and outdoor environmental data be useful to help you managing asthma in your daily routine?

#### Artificial Intelligence (AI)

General perception. AI is a tool to get things done quickly, it is great! We need to move from algorithm-based AI to enabling AI to learn and make recommendations, which would enable personalised advice. However, AI could be potentially harmful if no human checks are in place, so human checks are required in the system.

Specific points raised by the PPI colleagues:

- AI should be tailored to individuals. It should be able to collect the right information for accurate predictions.
- AI should be able to learn about the individual’s daily routine and be able to interact with a person in their daily life.
- AI should interact with the smart technology people are currently using at home.
- AI needs to learn about the individual to build in the unique human elements.
- AI needs to know whether its advice is useful or not to individuals. Individuals would like to be able to tell the AI if they will act on the information or not, so that the AI learns about individual preferences.
- AI should have a supportive role (not just monitoring) to help an individual understand something or remind them of something, and the rationale for the advice or recommendation.
- AI should observe information for a long enough time period to include seasonal cycles of triggers.
- AI and any other mechanical and digital measurement devices would need to be supplied for low-income individuals to make it accessible.

PPI colleagues suggested AI could be a useful tool for identifying triggers and the causes of asthma symptoms; to predict and advise on pollution levels that could exacerbate a person’s asthma. AI could help correlate symptoms or peak flows with changes in exposure to triggers which could then help with trigger avoidance. Reminders about practical tasks (such as cleaning a spacer device) would be useful. It would be useful if AI could determine an individual’s asthma status and provide nudges and prompt advice on stepping up or stepping down medication. In the event of an attack, it would be helpful if AI could advise what to do.

Importantly, the AI system has to be connected with the patient’s GP/asthma nurse. However, PPI colleagues worried about the communication logistics such as what would the practices do after receiving the data, would they change the medication treatment only based on the data without discussing with the patient. The PPI colleagues considered that it would be good to use the data as a trigger to arrange a routine review, while using the AI to continue collecting their asthma data to inform the consultation.

#### Indoor and outdoor environmental data

Environmental data needs to be local to the individual if it is to detect useful changes in air quality. The air quality information should be plotted on the same graph as asthma control, so they can correlate air quality levels with control. The data displayed could be all available environmental data, or just those components that the AI has determined is a trigger for the individual. One volunteer suggested that if they knew that air quality was good or local pollen levels were falling, they would feel confident to reduce their medication use.

There was some debate about the value of air quality monitoring. One volunteer noted that they could not necessarily do anything about the pollutants: for example, if they wanted to go to a cinema, they would still go even if the indoor air quality was poor. If they rent accommodation in a polluted area, they still had to live there. Conversely, another volunteer considered that ‘knowledge sets people free’. They wanted to have the information so they could make decisions by balancing the risk and benefits of increasing their exposure to triggers, for example, by exercising outdoors when pollen levels were high.

### Feedback on exercise 2: the three potential use cases of AI, indoor and outdoor environmental data

**Fig 1:**
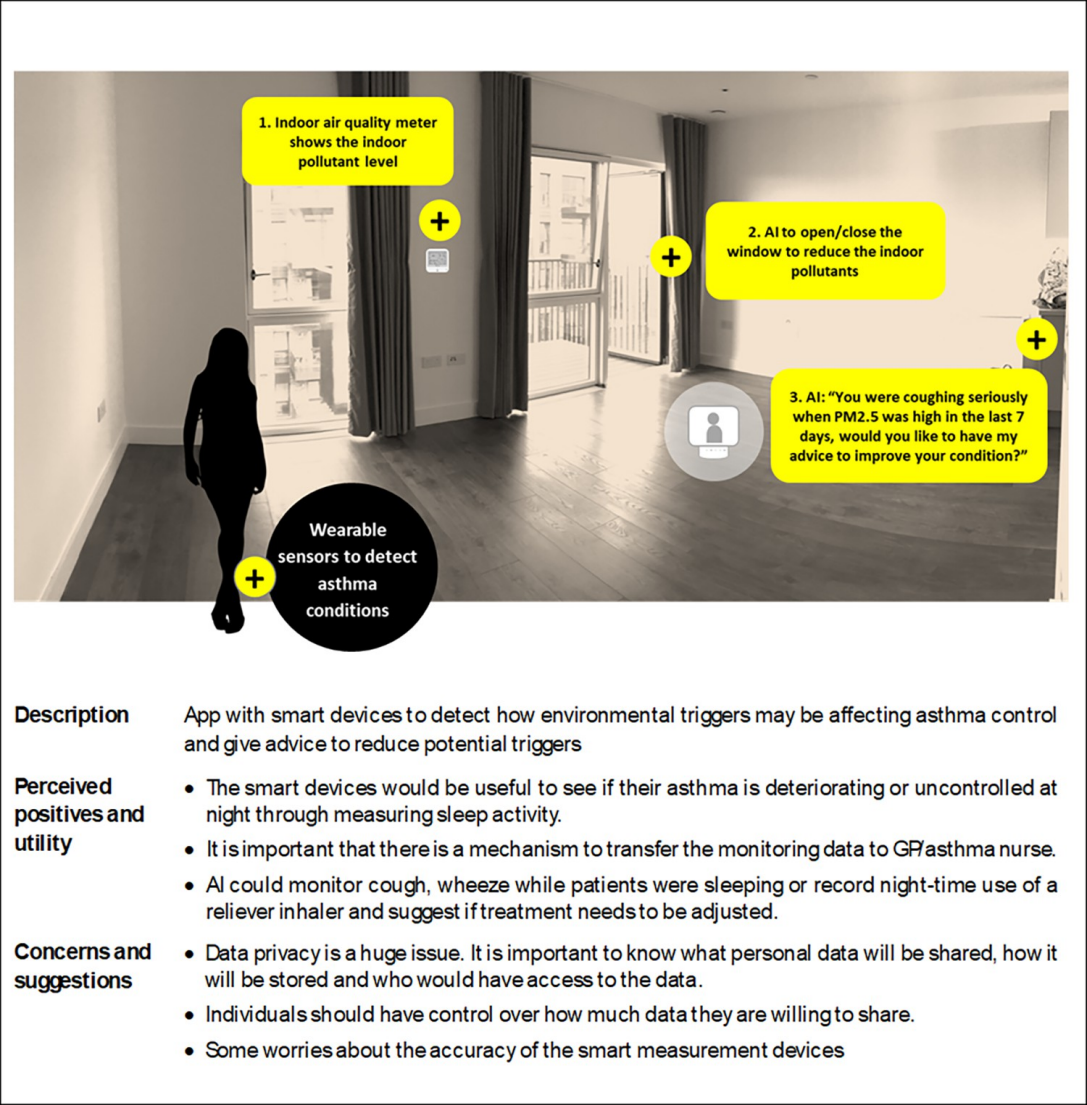
User case 1: An asthma connected system.

**Fig 2:**
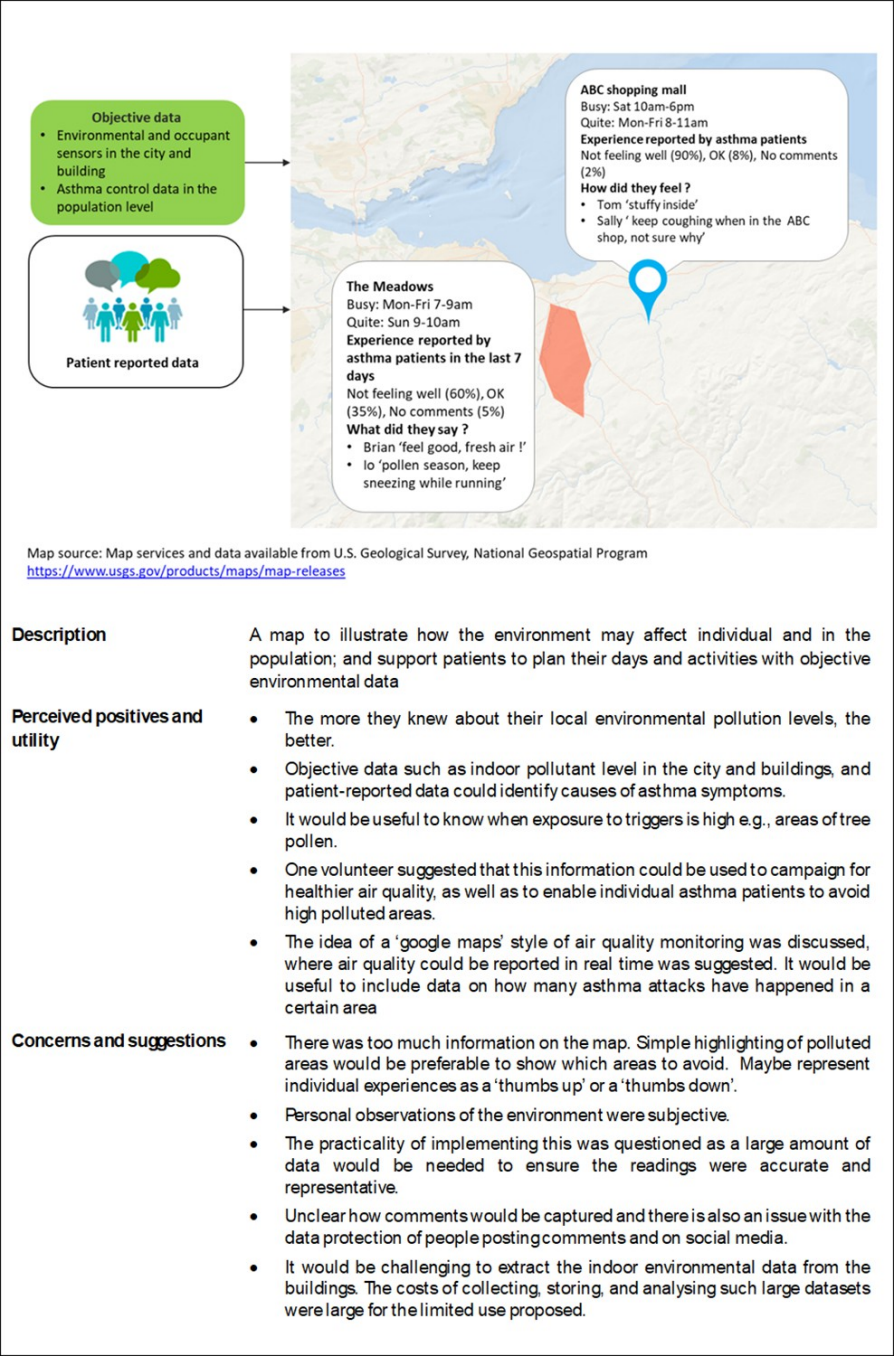
Use case 2: An open asthma map.

**Fig 3:**
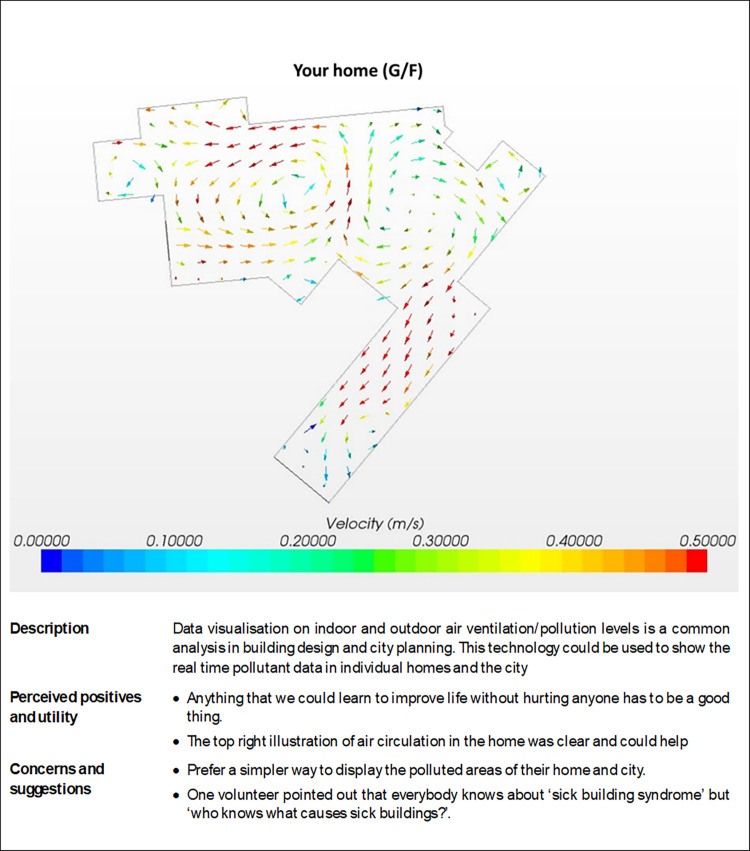
Use case 3: Data visualisation.

The illustrations used to introduce the use cases in the workshops/discussions are illustrated in Figs 1–3, along with the positive and negative comments and suggestions.

*a) A connected system*, *including an app with smart devices to detect how environmental triggers may be affecting asthma control and give advice to reduce potential triggers*.

PPI colleagues expressed that the smart devices would be useful to see if their asthma is deteriorating or uncontrolled at night through measuring sleep activity. It is important that there is a mechanism to transfer the monitoring data to GP/asthma nurses.

AI could monitor cough, wheeze while patients were sleeping or record night-time use of a reliever inhaler and suggest if treatment needs to be adjusted.

Concerns: PPI colleagues stated that data privacy is a huge issue. It is important to know what personal data will be shared, how it will be stored and who would have access to the data. Individuals should have control over how much data they are willing to share. Some PPI colleagues commented on a worry about the accuracy of the smart measurement devices.

*b) An open asthma map to understand how the environment may affect individual and in the population; and support patients to plan their days and activities with objective environmental data*.

The PPI colleagues thought that the more they knew about their local environmental pollution levels, the better. It would be useful to use objective data such as indoor pollutant level in the city and buildings, and patient-reported data to identify causes of asthma symptoms. They would also find it useful to know when exposure to triggers is high e.g., areas of tree pollen. One volunteer suggested that this information could be used to campaign for healthier air quality, as well as to enable individual asthma patients to avoid high polluted areas.

The idea of a ‘google maps’ style of air quality monitoring was discussed, where air quality could be reported in real time was suggested. PPI colleagues suggested it would be useful to include data on how many asthma attacks have happened in a certain area.

Some PPI colleagues suggested there was too much information on the map. Simple highlighting of polluted areas would be preferable as they were only interested in what areas to avoid.

One volunteer was concerned about the personal observations of the environment which were seen to be subjective. Another volunteer suggested representing individual experiences as a ‘thumbs up’ or a ‘thumbs down’. It was also unclear how comments would be captured and there is also an issue with the data protection of people posting comments and on social media.

Another volunteer questioned the practicality of implementing this intervention as a large amount of data would be needed to ensure the readings provided by other asthma sufferers were accurate. They also believed that would be challenging to extract the indoor environmental data from the buildings (e.g. the shopping mall shown in this use case). The costs of collecting, storing, and analysing such a large dataset were considered too large for the limited use proposed.

*c) Data visualisation on indoor and outdoor air ventilation/pollution levels is a common analysis in building design and city planning*. *This technology could be used to show the real time pollutant data in individual homes and the city*.

Most PPI colleagues preferred to have a simpler way to display the polluted areas of their home and city. One volunteer pointed out that everybody knows about ‘sick building syndrome’ but ‘who knows what causes sick buildings?’. They appreciated that anything that we could learn to improve life without hurting anyone has to be a good thing. They thought the top right illustration of air circulation in the home was clear and could help.

#### Follow up

All PPI colleagues wanted to stay connected with the C4A project and five were keen to be authors on a paper reporting the outcomes of the workshops.

## Discussion

The workshop discussions with PPI colleagues provided advice on the technology features we should include in our next digital intervention and the AI-related and the research topics that we should pursue. Their advice will help ensure that our investigation will be useful to asthma patients, and that the technology will be implementable in the real-world healthcare services. This is a novel and practical approach to addressing the gap between different phases of the development cycle of healthcare technologies, merging past research evidence, preferences, and practicalities, as well as shifting the focus from researcher-driven to a patient-centric approach.

### Informing the approach to interacting with AI in our next digital intervention

There was general agreement that AI does not replace humans. PPI colleagues wanted AI to support data collection, remind them about self-management tasks, teach them about asthma triggers, identify risk, and empower them to confidently look after their asthma. Echoing concerns from previous studies [17,20,21], they were clear that:

- Clinician support is needed to check the AI decision and be available for consultation when required. Patients trust that AI can support their self-management if clinicians are involved in the decision loop.
- The patient has to actively teach the AI (as opposed to only providing data to train the AI). A mechanism is needed for patients to teach the AI what is right or wrong about their asthma. For example, AI will ask for feedback on their decision, patient will give a positive credit score to the AI on right decision and negative for a wrong decision.
- The patient must make the final decision whether to follow the advice of the AI–and to tell the AI if they like their advice or not.

In our next digital intervention, we will consider the AI techniques in generative AI Large Language Models (LLM), Large Action Model neuro-symbolic models, and Human-in-the-loop (HITL) ensemble learning model to set up the AI [22,23,24]; with an aim to incorporate the patients and clinicians in the AI decision loop to support asthma self-management, and we will test and verify the user’s trust, satisfaction, and asthma outcomes of such system.

### Informing the technology features in our next digital intervention

The PPI colleagues had clear preferences about the features they wanted to see in future technology:

- Correlating asthma symptoms and environmental data to identify potential triggers and providing self-management advice personalised to their daily routine,
- There was interest in illustrating areas with poor air quality on a real-time asthma map or mapping poor air quality at home—though avoiding over-complex displays. The experience of other people with asthma in the area could be useful, but comments would need to be moderated.
- Seasonality was noted as important, and data collection should extend over a seasonal cycle to see changing triggers and associated patterns of asthma symptoms.
- Connectivity was essential–transferring monitoring data and facilitating necessary contact with clinicians, connecting with the smart devices in the home.

PPI colleagues’ voiced concerns about data privacy and security of the system, especially worries about gathering information in public areas, and the permissions required to link the C4A system with indoor environmental data from existing building management systems used in most public buildings (shopping malls, offices, universities, hospitals). Worrying that personal data may be collected by third parties without any consents, resonated to the ethical concerns from stakeholders in many of the AI healthcare applications [25].

In our next intervention, we will incorporate these features into our existing C4A connected app in phases, involving PPI colleagues to contribute their thoughts on prototypes to ensure the interface is built to be useful, and simple to understand and use. There are multiple asthma sensors meeting regulatory standards to which we can link to build a connected system [17], and we will link where possible with existing environmental systems [26]. The system must comply with the General Data Protection Regulation (GDPR) and to connect with the smart build environment system that has implemented the data security strategies to safeguard patients’ personal data [27].

### Informing research topics

Patients want to avoid triggers that affect their asthma, but a threshold is required to alert or highlight an area as having poor air quality. There are existing guidelines and regulations defining acceptable air quality thresholds that the system could adopt. For example, WHO set the acceptable threshold of 24-hour mean PM2.5 at 15ug/m3 in 2021[4]. Various building and ventilation regulations use different thresholds [28]. Some patients tend to read the actual air quality reading on the device and use their past experience to decide whether they should take avoiding action. Furthermore, as discussed in the workshops, it may not be possible to take avoiding action if the patient lives in a polluted area. McCarron et al research suggests personalised air quality data and greater public engagement with these data will together encourage patients to minimise their exposure to pollutants [29].

In our ongoing research, we will investigate the best practical and safe thresholds for people with asthma, and use these to inform AI alerts, and what practical and safe self-management advice should be offered. There will also be the potential to develop public engagement activities that could encourage people with asthma to avoid indoor and outdoor air pollutants [30].

### Health equity

PPI colleagues recommended that low-income patients should be provided with free smart devices. This recommendation has two implications for the research team. The cost of the device must be incorporated in the budget for future grant proposals, and when planning the implementation, we need to collaborate with cross-disciplinary experts in health equity to explore how a service can be equitable, ethical, open and sustainable.

### Liability

PPI colleagues recommended involving clinicians to check the AI decision and are not confident to follow unsupervised AI advice. The extent to which clinicians, manufacturers and patients are liable for incorrect advice given by AI will need to be resolved. There are many reasons for incorrect advice: an incorrect algorithm, inaccurate calibration of a devices/sensor, software ‘bugs’, or incorrect interpretation by the user (patient/clinician)[31]. In our next intervention, we will need to identify the user interface characteristics that relate to the safe use/generation of advice, by involving the clinician in the decision loop. We need to map and test the interaction model between AI, patient and clinician conforming to standards set by IEC 62366.

## Reflections, challenges, and limitations

The PPI members had diverse experiences and knowledge of technologies, but they all had an interest in asthma self-management technologies. We recognise that they may not represent the general population. However, their discussion on the use cases has given us feedback on what they want to see on an interface. They were willing to express both positive and negative views, as they were aware that this would support the researcher to shape a future technology. Resonating with the PPI involvement in developing digital technologies, this is helpful to resolve the problem where participants are reluctant to criticise an existing technology in research [32].

We provided both in-person and online options and many PPI colleagues were happy to take part in remote online discussion rather than travelling to the University campus. We eventually conducted one in-person and three online workshops. The discussions in the online and in-person workshops were similar, though it was challenging for the researcher to capture in-depth views during online workshops because of the lack of the body language, especially if the PPI colleagues were in off-camera mode due to personal preference.

The researcher who led the workshops had a background in technology and population health science. C4A was developed in her PhD project, and she had conducted the previous mixed methods studies that explored preferences for asthma self-management technologies. She was thus able to bring previous patients views to the discussion which encouraged a wide variety of discussion in the workshops, though may also have influenced the themes. The facilitators were early career researchers and a research administrator experienced in supporting PPI, therefore were adept at raising questions in the workshop when they observed PPI colleagues were confused by some technology jargon.

## Conclusion

There is lack of literature on how PPI can shape a digital intervention study. Our experience suggests that PPI advisory workshops can support a shift from the traditional user role of a tester of a digital intervention, to a co-design partner who helps shape the next iteration of the intervention. It is known that those who live with a condition such as asthma on a day-to-day basis are best placed to have an awareness of the tools that might assist them in avoiding exacerbations and minimising asthma attacks. Their involvement throughout the research and development process are essential to ensure that the future tools deliver worthwhile benefits. PPI involvement allowed the researcher to collect both positive and negative feedback from the patients, which provided a direction for the design of a digital intervention that is useful for patients in their self-management.

## Supporting information

S1 Table Exercise 1 (for participant with asthma)—My current self-management routine. (DOCX)
