# Supplementary material for: Patient and public involvement workshop to shape artificial intelligence-supported connected asthma self-management research
Source: PLOS Digit Health. 2024 May 30;3(5):e0000521. doi: 10.1371/journal.pdig.0000521 (PMC11139256; doi:10.1371/journal.pdig.0000521)
Supplement: S1 Table — (DOCX) [file pdig.0000521.s001.docx]

**Aim of exercise one:** We would like to understand your current self-management routine, and how do you think indoor and outdoor air data, and AI can be useful in your daily routine. Therefore, we are hoping you could complete the table below and present your responses in the virtual workshop.

Fonts highlighted in grey below give some examples on how to complete the table. Any questions, please do not hesitate to let us know. Thank you.

| S1 Table: Exercise 1 (for participant with asthma ) - My current self-management routine | | | | |
| --- | --- | --- | --- | --- |
|  | 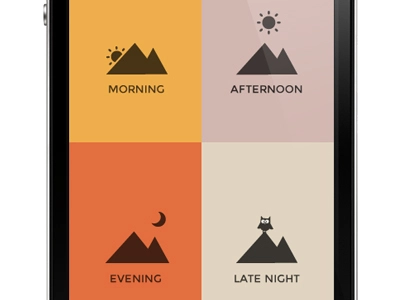 | 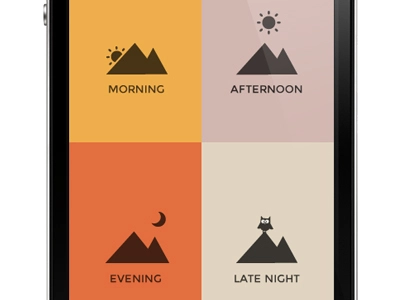 | 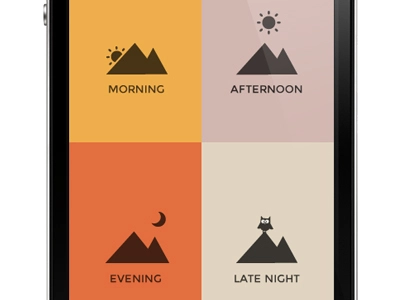 | 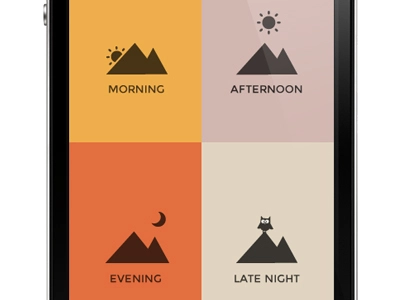 |
| **Scenario** | 6am-12:00 noon | 12:00 noon-5pm | 5pm-9pm | 9pm-4am |
| 1. Everyday, at these times I think about or monitor my asthma by… | e.g.  - taking 2 puffs of my brown inhaler | e.g.  - N/A | e.g. taking 2 puffs of my brown inhaler | e.g. checking the met office app about the air pollution level |
| 2. When I feel my asthma is getting worse, I would like to be able to… | e.g.  - measuring my peak flow | e.g.  - doing less exercise | e.g.  - avoiding the crowd when I am going back home | e.g.  - measuring my peak flow |
| 3. When I want to know what is affecting my asthma, I would like to be able to … |  |  |  |  |
| 4. When I want to avoid my asthma triggers, I would like to be able to … |  |  |  |  |
| 5. In the future, I believe the air indoor and outdoor air quality monitors and AI can help me look after my asthma by… |  |  |  |  |
